# Challenges in Pedicatric Orthodontics and Radiology: Evidence from Recent Studies and Implications for Clinical Practice: A Narrative Review

**DOI:** 10.31661/gmj.v13iSP1.3733

**Published:** 2024-12-30

**Authors:** Yasaman Nakhaei, Faranak Farahmand, Mohammad Hossein Yazdanpanah, Nastaran Saeidi, Seied Kaveh Ghaffarzade, Sahar Soleimani, Farnaz Haji Abbas Oghli

**Affiliations:** ^1^ Department of Pediatrics, Faculty of Dentistry, Mashhad University of Medical Sciences, Mashhad, Iran; ^2^ Department of Pediatric Dentistry, School of Dentistry, Shiraz University of Medical Sciences, Shiraz, Iran; ^3^ Dental Research Center, School of Dentistry, Shiraz University of Medical Sciences, Shiraz, Iran; ^4^ Islamic Azad University of Medical Sciences Department of Dentistry, Tehran University of Medical Sciences, Tehran, Iran; ^5^ Department of Prosthodontic Dentistry, School of Dentistry, Azad Tehran University of Medical Science , Tehran, Iran; ^6^ Department of Pediatric, Faculty of Dentistry, Tehran Medical Sciences, Islamic Azad University, Tehran, Iran

**Keywords:** Pediatric Dentistry, Dental Radiology, Orthodontics, Radiation Exposure, Technological Advancements, Patient Cooperation, Safety Measures

## Abstract

Background: The pediatric population presents unique challenges in dental and maxillofacial radiology and orthodontics. This review aims to highlight the current challenges and explore emerging solutions in pediatric dental radiology and orthodontics. Materials and Methods: A comprehensive review of recent literature was conducted to synthesize findings on pediatric dental radiology, orthodontic appliances, and patient management. Results: Technological advancements, including pre-orthodontic trainers, clear aligners, 3D printing, and AI-driven tools, enhance early intervention, hygiene, precision, and personalized treatment planning. AI models for tooth numbering, detection, and segmentation on panoramic radiographs show high accuracy. Radiographic techniques like CBCT and panoramic tomography are effective for identifying dental issues such as crowding, delayed eruption, impaction, and ectopic eruption, with AI-assisted prediction and deep learning approaches offering promising solutions. CBCT is preferred for diagnosing mandibular asymmetry, but orthopantomography is advisable as a first-line diagnostic tool due to lower radiation exposure. Effective patient cooperation is enhanced through communication, positive reinforcement, and parental involvement, with techniques like the “tell-show-do” method and visual aids improving compliance and reducing anxiety. Innovations like open MRI designs, noise reduction, and virtual reality sessions enhance comfort and cooperation during exams. Given children’s higher radiosensitivity, strict adherence to dose reduction protocols, the ALARA principle, and effective communication with families are crucial for managing radiation risks. Conclusion: Ongoing research and education are essential to ensure optimal care and safety for radiology practices for pediatric patients.

## Introduction

The pediatric population presents unique challenges due to the ongoing development of their dental and maxillofacial structures, which can lead to variations in the appearance of radiographic images [1]. Understanding these differences is essential for accurate diagnosis and treatment planning, as the anatomy of children’s teeth, jawbone, and surrounding tissues differs significantly from that of adults [2]. For instance, the dental follicle, a normal structure in pediatric panoramic radiographs, must be distinguished from underlying pathologic conditions [1]. According to Campbell et al. [3], valid reference values for intraoral radiographs in children and adolescents are still missing, and further studies are necessary to determine the meaningful dose reference level for children. The use of digital radiography is almost ubiquitous, but the use of rectangular collimation is limited [4][5]. To reduce radiation exposure, dentists treating children should be familiar with radiation exposure guidelines and consider using dose-reduction strategies recommended by the Image Gently Campaign in Dentistry [5]. Motion artifacts in pediatric dental radiology are a significant concern, as they can compromise image quality and lead to repeated scans, increasing radiation exposure [6][7]. A study found that motion artifacts were nonsignificantly different between 0.55 T MRI and ultra-low-dose CT [ULD-CT] in pediatric patients with supernumerary and ectopic teeth [6]. Another study reported that repeated scans due to motion artifacts occurred in 1.96% of patients undergoing cone-beam computed tomography [CBCT] scans [7]. The movement characteristics of young patients can significantly impact CBCT image quality, with lower image quality observed when movement occurs several times, has a long duration, or is multiplanar [8]. To minimize motion artifacts, high scanning speed and a half rotation (180°) can be used to reduce radiation dose and motion artifacts [9]. Overall, pediatric orthodontics and radiology are characterized by complex challenges that require a careful balance of patient needs, ethical considerations, and practical constraints. By examining these issues in depth, this review seeks to illuminate current challenges and explore emerging solutions that may shape the future of care in these fields.

## Technological Advances and Limitations

Recent innovations in orthodontic appliances for children, such as pre-orthodontic trainers, have shown success in early intervention for conditions like Class II malocclusions [10]. Clear aligners have become a popular child-friendly alternative to traditional braces, offering benefits like improved hygiene and aesthetics, though they are best suited for mild dental misalignments [11]. Advancements in digital technology and AI, including 3D printing, enable the creation of highly customized appliances, enhancing precision and reducing turnaround times, especially beneficial for children with complex conditions like cleft lip and palate [12][13]. Novel software and AI-driven tools facilitate detailed phenotyping and personalized treatment planning, marking a shift toward "precision orthodontics" [14]. Studies also highlight that visually appealing appliances, such as vivid pedo appliances, significantly improve children’s compliance and acceptance, emphasizing the importance of engaging children in their treatment [15].

The integration of artificial intelligence (AI)in dental radiography has shown great promise, with AI models being developed for tooth numbering and detection using dento-maxillofacial radiographic images [16]. Furthermore, the use of direct digital radiography has been found to be effective in detecting proximal caries in primary teeth, highlighting the potential of digital technologies in pediatric dental care [17]. Additionally, studies have emphasized the importance of customizing dental tools and technology for children, underscoring the need for ongoing education and advocacy to ensure that dental professionals have access to the best tools for treating young patients [18]. The application of three-dimensional (3D)printing in pediatric dentistry has also been explored, with promising results [18].

## Pediatric dental radiology and Malocclusions

Research has shown that radiographic characteristics in the mandibular condyles of orthodontic patients can be indicative of malocclusions, with studies suggesting that approximately 2.2% of children exhibit such characteristics [19]. Furthermore, the use of cone-beam computed tomography (CBCT)has been found to be effective in evaluating transverse maxillomandibular discrepancy and dental compensation in children with skeletal Class III malocclusion [20]. In the context of Class I Malocclusion, radiological variations in mandibular condyles have been observed, highlighting the importance of thorough radiographic examination [21].

Initially, broad screenings of malocclusions in the 1970s and 1980s laid the groundwork for systematic orthodontic examinations, emphasizing the early identification of malocclusions. A Finnish study underscored the importance of ongoing check-ups by demonstrating that orthodontic conditions diagnosed at age 7 often persisted into adolescence [22]. This early and continuous monitoring is crucial because studies have shown that when initial diagnoses do not accurately reflect patient needs, there is a higher likelihood of unsatisfactory orthodontic results, particularly in complex cases involving skeletal discrepancies or severe malocclusions [23]. For example, a lack of precise diagnosis can lead to inappropriate choices between orthodontic camouflage and surgical intervention for conditions like Class II malocclusion, affecting both aesthetics and long-term functional stability [24]. Radiology plays a pivotal role in this diagnostic process. Advanced imaging techniques, such as cone beam computed tomography (CBCT), provide detailed 3D images of the craniofacial structures, enabling orthodontists to accurately assess skeletal relationships, tooth positions, and airway dimensions [25]. This level of detail is essential for making informed decisions about treatment plans, especially in complex cases. For instance, CBCT can help identify early signs of skeletal Class III malocclusion, allowing for timely intervention and better outcomes [26]. Patient satisfaction with orthodontic outcomes is often tied to how well treatment aligns with initial expectations and perceived functional improvements, both of which are rooted in accurate early assessments [27]. This is particularly important for conditions such as skeletal Class III malocclusion, which, when treated alongside early childhood caries, requires a multi-phase approach that incorporates caries management, behavioral modifications, and orthodontic intervention to prevent more severe malocclusions from developing [27][28][29]. The importance of early and accurate referral decisions is underscored in Batarse et al. (2019), where pediatric dentists were found to have higher referral rates for complex cases, helping to ensure that children receive specialized care when needed [30]. Radiological assessments are often a key factor in these referral decisions, as they provide critical information about the severity and nature of the malocclusion [31]. For conditions like Class II malocclusion, Batista et al. (2018) noted that while early treatment could reduce trauma risks, it may not significantly alter long-term outcomes compared to treatment initiated in adolescence, suggesting that complex cases require careful assessment of timing and potential benefits [31]. Radiology can provide valuable insights into the growth patterns and skeletal maturity of the patient, helping orthodontists determine the optimal timing for intervention [26][27][28][29][30][31]. Innovative techniques, such as the orthotropic approach, aim to prevent complex malocclusions early by guiding jaw and airway development [31]. Radiological imaging is crucial in monitoring the progress of these treatments, ensuring that the jaw and airway are developing as expected [27][28]. Effective interdisciplinary approaches, such as those demonstrated by Liaw et al. (2021) in the treatment of a complex Class III malocclusion, illustrate how collaboration among orthodontists, implant specialists, and surgeons, supported by advanced radiological techniques, facilitates optimal aesthetic and functional outcomes [29].

## Pediatric Radiology of Dental Crowding

The diagnosis of dental crowding in children often involves the use of radiographic imaging, such as panoramic tomographs, to assess the position and development of teeth [32]. This approach can help identify potential issues early on, allowing for timely intervention and treatment. In addition, the use of radiographic analysis, such as panoramic tomographs, has been found to be effective in identifying dental crowding and other developmental issues in children [32]. Predictive models have also been developed to forecast changes in incisor and canine crowding, taking into account factors such as dental arch form and tooth size [33].

A prospective study evaluated the accuracy of a semi-automatic 3D digital setup in predicting the outcome of orthodontic treatment with fixed appliances for moderate crowding correction, revealing that while the average deviations were less than 1 mm, individual case disparities were significant [34]. A study developed machine learning models to predict extraction or non-extraction decisions in orthodontic treatments of dental crowding, achieving high accuracy with logistic regression [35]. Additionally, research has also focused on the application of AI in predicting pubertal mandibular growth, which is crucial in predicting dental crowding in pediatric patients [36][37].

## Pediatric Radiology of Tooth Eruption Disorders

Radiographic features such as delayed eruption, impaction, and ectopic eruption can be identified, allowing for early intervention and treatment [38][39]. The use of 3-D imaging techniques, such as cone-beam computed tomography, can provide detailed information on tooth morphology and eruption patterns, enabling accurate diagnosis and treatment planning [39][40]. Furthermore, radiographic assessment of dental anomalies, including crown and root malformations, agenesis, and eruption deviations, can help identify potential complications associated with tooth eruption disorders [41][42][43]. By utilizing radiology, dental professionals can develop effective treatment strategies to address tooth eruption disorders and prevent long-term consequences.

Studies have shown that AI-assisted radiographic prediction of lower third molar eruption can be a valuable tool in determining the timely extraction of these teeth [44]. The use of deep convolutional neuronal networks has been explored for the automatic detection of periapical osteolytic lesions on cone-beam computed tomography [44]. Furthermore, AI-driven tools have been developed for tooth detection and segmentation on panoramic radiographs, demonstrating high accuracy and consistency [45]. These tools have the potential to facilitate and optimize dental care by providing fast and accurate measurements of molar angulations on panoramic radiographs [46]. The application of AI in radiology has also been extended to the detection of ectopic eruption of maxillary first molars, allowing for earlier detection and timelier intervention [46][47].

## Mandibular asymmetry

Mandibular asymmetry refers to the dimensional differences between the left and right sides of the mandible in terms of size, form, and volume, which can result in problems with functionality and appearance [48]. According to Liukkonen et al. [49], the prevalence of mandibular asymmetry in children can be measured using orthopantomograms, which provide a two-dimensional representation of the mandible. However, Bakri et al. [50] suggests that cone beam computed tomography (CBCT) is the preferred examination method for diagnosing mandibular asymmetry, as it allows for the assessment of a 3D structure with a 3D image. Nevertheless, the use of orthopantomography as a first-line diagnostic tool in children is advisable due to less radiation exposure [48]. The diagnosis of mandibular asymmetry in children is essential, as early intervention can help address the condition and prevent further complications [51]. Therefore, a comprehensive radiologic assessment, including CBCT and orthopantomography, is necessary for accurate diagnosis and treatment planning. New analysis method of digital panoramic radiographs has been developed to differentiate between functional and morphological mandibular asymmetry in children with and without unilateral posterior crossbite [50][51]. Recent study have investigated the use of multilayer panoramic radiography, a new tool that has shown similar performance to conventional panoramic radiography in the evaluation of mandibular third molars [52].

## Radiographic assessment of dental anomalies

The use of advanced imaging techniques such as panoramic radiography, computed tomography (CT) scans, and magnetic resonance imaging (MRI) has improved the diagnosis and treatment of dental anomalies in children [53][54]. A study by Jaber et al. [55] demonstrated the effectiveness of orthopantomography in diagnosing condylar bone pathology in patients with temporomandibular joint disorders. Another study by Wan et al. used MRI to quantify the signal intensity ratio in the diagnosis of temporomandibular condylar resorption in young female patients. The morphology of vascular ring arch anomalies, which can be detected using CT or MRI scans, influences prognosis and management [56]. Additionally, the molar tooth sign, which can be detected on axial brain MRI, is a characteristic feature of Joubert syndrome and other distinct syndromes [53]. The use of deep learning-based approaches has also shown promise in the detection and classification of dental structures and treatments on panoramic radiographs of pediatric patients [55].

## Radiology role in orthodontic treatment planning

Recent studies underscore the complexities of using advanced radiological guidance in orthodontic treatment planning, especially as newer imaging technologies like CBCT, MRI, and 3D digital planning tools are integrated. The development of clinical guidelines remains essential, as the variability in radiation exposure and diagnostic effectiveness of various imaging methods (e.g., CBCT, lateral cephalograms, and OPG) presents challenges for standardization. Kapetanović et al. (2020)and Storozhchuk & Mykhalchuk (2023) propose guidelines and algorithms to improve radiological examination efficiency while minimizing repeat exposure, especially in pediatric patients, who are more vulnerable to radiation risks [56][57][58]. Despite CBCT’s advanced diagnostic capabilities, such as providing clear views of pharyngeal airway structures and impacted teeth, studies by Abdelkarim (2019)and Cesur & Orhan (2021) highlight the concerns with increased radiation and limited accessibility in certain regions, which complicates widespread adoption [59][60].

The digital workflow in orthodontics, particularly for complex treatments involving aligners and implant-supported devices, is enhanced by advanced 3D planning but demands precise, interdisciplinary collaboration. Techniques like digital aligner planning combined with CBCT enable the pre-positioning of implants as skeletal anchors, improving treatment stability as described by Kirlys et al. (2022) [61]. Furthermore, studies such as Brugnami et al. (2021) and Nawrocka et al. (2022) explore 3D imaging for complex Class III cases and interdisciplinary treatment of odontogenic cysts, respectively, demonstrating the potential for precision but highlighting the need for reliable soft tissue mapping to complement the skeletal data provided by CBCT [62][63]. Collectively, these studies illustrate how radiological guidance, while invaluable for precise treatment planning, requires careful consideration of patient safety, accessibility, and technological constraints, which remain crucial challenges for orthodontic practice.

## Patient Cooperation and Management

Managing cooperation and compliance in pediatric orthodontic patients can be one of the most challenging aspects of treatment, requiring a combination of effective communication, behavioral strategies, and a supportive treatment environment. Studies have shown that patient cooperation is influenced by several factors, including age, parental involvement, and the perceived importance of the treatment. For instance, younger children generally exhibit less cooperation with orthodontic devices due to their limited understanding and tolerance, while older children, especially teenagers, may resist treatment due to esthetic concerns, discomfort, or lack of motivation [64][65][66][67]. Parental involvement is often crucial; children are more likely to comply when parents are actively engaged and provide encouragement throughout the treatment process.

Effective strategies to enhance compliance include using behavior management techniques such as positive reinforcement, reward systems, and clear, age-appropriate explanations about the importance of treatment. Research has highlighted the importance of fostering a collaborative relationship between the orthodontist, the child, and the parents. For instance, a study by Staines et al. (2019) found that there was often a discrepancy in how behavior was perceived by dentists, parents, and children, with guardians generally rating their child’s behavior more favorably than clinicians [68]. Clear communication is vital, and techniques such as the "tell-show-do" method, where the orthodontist explains and demonstrates the procedure before performing it, can reduce anxiety and improve cooperation. For adolescents, engaging in discussions about treatment goals and the potential long-term benefits, as well as using reminders like text messages or emails, has been shown to significantly improve compliance with fixed orthodontic treatments [69]. Thus, the key to managing compliance lies in understanding the individual needs of the patient, maintaining consistent communication, and applying tailored behavioral strategies to foster motivation and adherence to treatment.

## Techniques for improving patient comfort during radiological exams

Improving comfort during pediatric radiological exams involves a combination of innovative technologies, specialized roles, and patient-centered approaches designed to reduce anxiety and enhance cooperation. One study highlights the use of "gentle touch" approaches such as room modifications, motion-corrected imaging, and parental involvement, which significantly reduce anxiety and discomfort in young patients [70]. In MRI, for instance, comfort is improved with open designs, noise reduction, and flexible radiofrequency coils, allowing children to feel less confined and anxious during scans [71]. Child life specialists are also crucial in these settings, as they provide pain management, distraction, and coping techniques that help children stay calm without the need for sedation, ultimately improving cooperation and imaging quality [72].

Additional strategies, such as customized positioning aids and comfort positioning, further enhance comfort and compliance during exams. Positioning aids tailored to pediatric needs, combined with caregiver presence, help create a supportive environment where children feel safer, though this raises considerations around caregiver exposure to scattered radiation [73]. Virtual reality (VR) has also proven effective in reducing anxiety, as VR sessions prior to chest radiography enable children to visualize the exam process in a calming, interactive way, thereby reducing distress and procedure time [74]. In addition, recent technological advances, such as low-tube voltage protocols in CT scans, significantly lower radiation doses, which not only improves safety but also enhances comfort by reducing the physical and psychological impact of the procedure on pediatric patients [75]. Together, these multifaceted approaches underscore the importance of combining technical innovations with compassionate, child-focused care to create a safer and more positive radiology experience for children.

Effective communication with young orthodontic patients and their guardians is crucial for enhancing compliance and ensuring successful treatment outcomes. Studies demonstrate that a combination of traditional and digital methods is especially effective. For instance, using email reminders with instructional video links has been shown to significantly reduce appliance breakage, particularly in patients from higher-income households who are accustomed to digital engagement [69]. Likewise, platforms like Instagram can reinforce chairside verbal instructions through multimedia content, enhancing young patients’ understanding of oral hygiene and increasing compliance [76]. Behavior change techniques, including motivational interviewing and digital reminders, also play a role in improving compliance, as these personalized approaches help patients feel more connected to their treatment and understand the importance of their own involvement [77]. Engaging adolescents presents additional challenges; a study found that young patients perceive risks differently, often underestimating the long-term impact of orthodontic care, which underscores the need for clinicians to explain risks and benefits in a relatable, immediate context [78].

Visual aids and supportive communication strategies also enhance the orthodontic experience. Simulation systems using 3D models have been shown to help young patients visualize treatment outcomes, which not only reduces anxiety but also aligns their expectations more closely with realistic outcomes [79][80]. Pediatric communication experts emphasize the importance of rapport-building, active listening, and encouraging decision-making to improve engagement and cooperation [81]. Moreover, studies have found that patients rate communication as more effective when clinicians encourage questions and use simpler language to make the treatment process more understandable [82]. Techniques like storytelling and playful dialogical approaches in oral health education have also shown positive effects, increasing enthusiasm and comprehension among pediatric patients [83]. The role of families in the decision-making process is highlighted in hypodontia treatments, where parents often defer entirely to clinicians. Studies indicate that family-centered communication tools could empower guardians, helping them actively participate in treatment decisions [84]. Together, these studies emphasize the value of a multifaceted communication approach that blends digital and in-person engagement, empathetic dialogue, and visual tools to foster understanding, support, and cooperation among young patients and their guardians.

## Radiation exposure in pediatric radiology

Radiation exposure in pediatric radiology remains a significant concern due to children’s higher radiosensitivity and longer life expectancy, which increases their susceptibility to radiation-induced health risks. Studies underscore the critical importance of reducing radiation doses in pediatric imaging, particularly in frequently used modalities like CT. For example, Goodman et al. (2019) emphasize the advancements in pediatric CT radiation safety and the need to balance diagnostic benefits with minimized exposure [85]. A survey by Ng et al. (2022) reveals a gap in radiation protection awareness among healthcare providers, calling for improved education on safety measures [86]. The risks associated with CT scans, including potential links to brain tumor development, are highlighted by Meulepas et al. (2018), who found an increased brain tumor risk with cumulative exposure, underscoring the need for strict adherence to dose reduction protocols [87]. Additionally, Abdou et al. (2021) stress the role of the imaging team in managing pediatric CT parameters effectively to achieve diagnostic quality at the lowest dose possible [88].

Effective communication with patients’ families is equally crucial in managing concerns over radiation exposure. Shah et al. (2023)report that nearly a quarter of parents are apprehensive about radiation risks, emphasizing the need for clear communication on the necessity and safety of imaging procedures [89]. Meanwhile, the application of the ALARA principle (As Low As Reasonably Achievable) is essential for safeguarding high-risk groups, as noted by Toma et al. (2019), who recommend dose-reduction strategies tailored for pediatric patients [90]. Furthermore, Aamry et al. (2020) discuss the variation in pediatric CTA doses across machines, underscoring the need for standardized protocols to ensure consistent and safe radiation exposure [91]. Finally, Paulson (2018) highlights the unique vulnerabilities of children during radiological emergencies, emphasizing the need for preparedness and protection strategies to limit radiation risks [92]. Together, these studies underscore the necessity of a multifaceted approach that combines technical advancements, effective communication, and rigorous safety protocols to minimize radiation risks in pediatric radiology.

Safety measures in orthodontic treatment are critical to protecting patients from complications such as dental decay, infection, and even systemic risks. A significant focus is on maintaining stringent oral hygiene to prevent the buildup of plaque around orthodontic appliances, as poor hygiene can lead to issues like periodontal disease and demineralization. Studies highlight that patients educated on oral hygiene by their orthodontists generally achieve better outcomes, suggesting that continuous education and monitoring are essential [93][94]. For patients with fixed appliances, additional cleaning devices and professional cleanings every few months can improve hygiene by reaching difficult areas [95]. Studies also emphasize that fixed appliances prompt patients to modify their routines, indicating a link between device type and compliance with hygiene protocols [96][97].

Infection control and patient-specific safety precautions are also vital in orthodontics, particularly for vulnerable populations. For example, patients at risk of infective endocarditis require additional precautions, as bacteremia from certain orthodontic procedures could increase their risk; collaboration with cardiologists and strict hygiene adherence are therefore recommended [98]. Additionally, research into micro-implant anchorage has shown promising results, as these devices enhance treatment efficacy and compliance in adolescents with minimal adverse effects, supporting their use as a safe orthodontic anchorage option [99]. To further reduce risks, aerosol-generating procedures in orthodontics are being minimized to protect both patients and clinicians from pathogen exposure, especially during pandemics [100]. Finally, the need for comprehensive guidelines to ensure safe and effective treatment in cases of maxillofacial deformities is underscored in recent reviews, highlighting that evidence-based standards are essential for maintaining patient safety in complex orthodontic procedures [101].

## Concslusion

In this narrative review, we have explored the multifaceted challenges faced in the fields of pediatric orthodontics and radiology, illuminating the pivotal role these specialties play in fostering the oral and craniofacial health of children. Pediatric orthodontics and radiology are not merely about correcting teeth alignment or producing diagnostic images; they are integral to early intervention strategies that address developmental anomalies, guide growth, and set a trajectory toward lifelong oral health.

Orthodontists and radiologists working with pediatric populations must navigate complex challenges ranging from managing patient compliance and ensuring safety in radiological practices to innovating with technology while safeguarding against its limitations. This review underscores the need for a judicious approach to imaging—balancing necessity against the risks of radiation exposure, especially pertinent in growing children. The discussion highlights advancements like low-dose imaging protocols and the adoption of non-ionizing imaging modalities which reflect an ongoing commitment to refine diagnostic practices and treatment modalities. Moreover, the issues of accessibility and socioeconomic barriers reveal that beyond clinical and technical challenges, broader systemic issues impact the delivery of care. These challenges necessitate a collaborative approach to health care, involving not only specialists but also family members and caregivers, to ensure comprehensive care that addresses all facets of a child’s development and well-being.

As we look toward the future, the ongoing advancements in technology and the increasing focus on early preventative care offer hope for addressing these challenges more effectively. However, the ethical considerations inherent in treating pediatric patients—balancing technological capabilities with patient safety and comfort—will continue to demand careful consideration and innovative solutions. In conclusion, pediatric orthodontics and radiology are dynamic fields characterized by both challenges and opportunities. By continuing to explore these issues and develop solutions that are both innovative and mindful of the unique needs of children, these specialties can significantly improve outcomes and contribute to the foundational health of future generations. This review calls for ongoing research, interdisciplinary collaboration, and policy support to overcome the barriers currently faced in these critical areas of pediatric healthcare.

## Conflict of Interest

The authors have no conflicts of interest relevant to this article to disclose.
